# Supplementary material for: Network models of protein phosphorylation, acetylation, and ubiquitination connect metabolic and cell signaling pathways in lung cancer
Source: PLoS Comput Biol. 2023 Mar 30;19(3):e1010690. doi: 10.1371/journal.pcbi.1010690 (PMC10089347; doi:10.1371/journal.pcbi.1010690)
Supplement: S1 Fig — (A) Overlap of individual PTMs that were more than 2.25-fold increased or decreased by different drugs, grouped by PTM type. (B) Density plots showing changes greater than 2.25 fold for each PTM and drug. Decreased PTM amounts in response to drug are highlighted in blue; increased amounts in yellow. Changes below the 2.25-fold threshold are omitted to highlight significant changes. (C) Correlation density between different PTMs on the same proteins for phosphorylation and acetylation; phosphorylation and ubiquitination; and acetylation and ubiquitination. Negative correlations selected for display as edges are highlighted in blue; positive correlations are highlighted in yellow. (PDF) [file pcbi.1010690.s001.pdf]

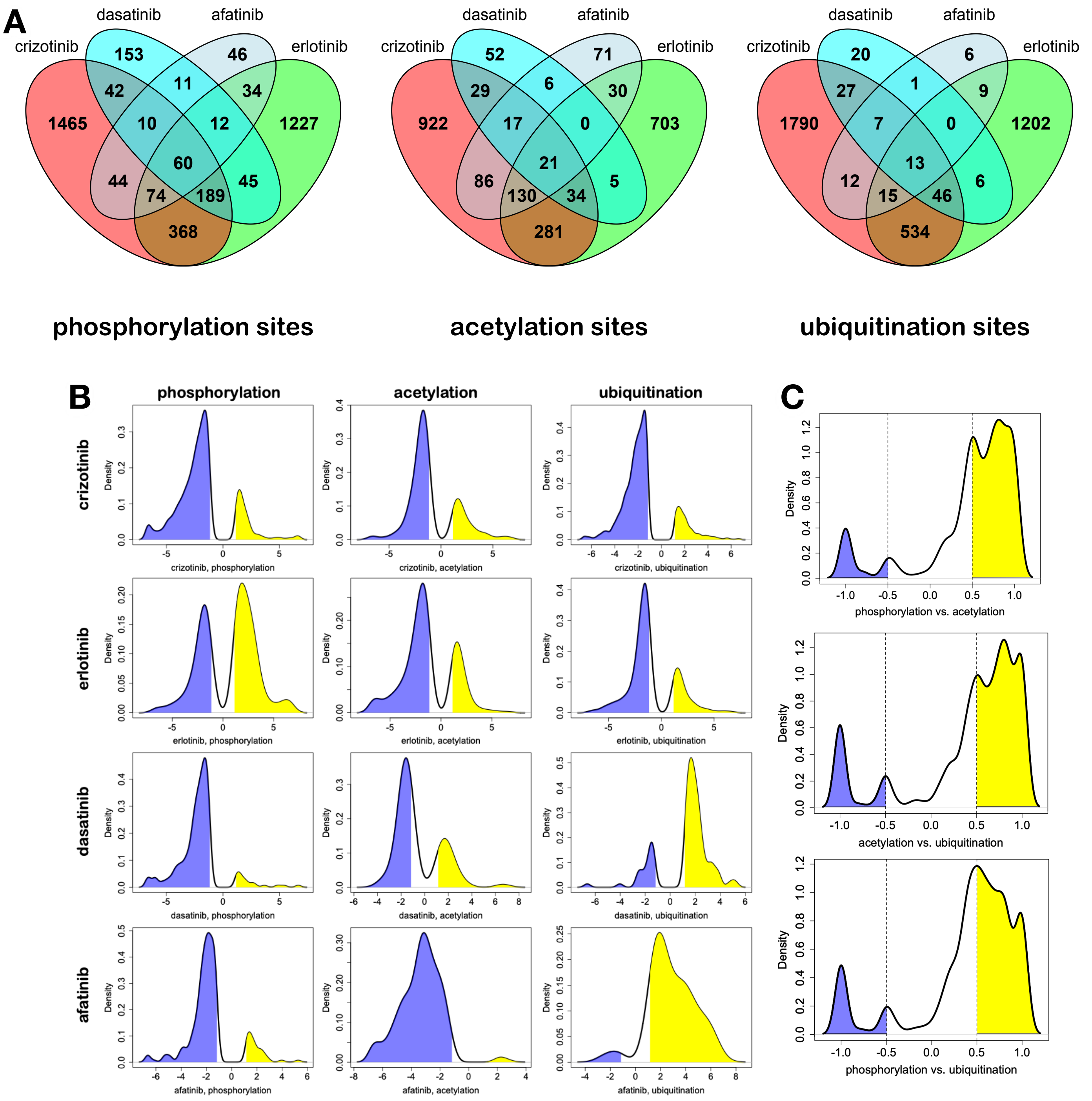

Figure S1. ***PTM changes in response to different TKIs.*** (A) Overlap of individual PTMs that were more than 2.25-fold increased or decreased by different drugs, grouped by PTM type. (B) Density plots showing changes greater than 2.25 fold for each PTM and drug. Decreased PTM amounts in response to drug are highlighted in blue; increased amounts in yellow. Changes below the 2.25-fold threshold are omitted to highlight significant changes. (C) Correlation density between different PTMs on the same proteins for phosphorylation and acetylation; phosphorylation and ubiquitination; and acetylation and ubiquitination. Negative correlations selected for display as edges are highlighted in blue; positive correlations are highlighted in yellow.
