# Supplementary material for: Network models of protein phosphorylation, acetylation, and ubiquitination connect metabolic and cell signaling pathways in lung cancer
Source: PLoS Comput Biol. 2023 Mar 30;19(3):e1010690. doi: 10.1371/journal.pcbi.1010690 (PMC10089347; doi:10.1371/journal.pcbi.1010690)
Supplement: S2 Fig — (A) Example three dimensional t-SNE embedding of PTM data using Spearman-Euclidean dissimilarity (SED) from PTM data. Co-clustered PTMs that are close to one another and share the same color. (B) CCCN of all PTMs. Yellow edges are positive correlation between co-clustered PTMs; blue edges are negative correlations, negative correlations < -0.5 among different PTMs on the same protein are included even if these PTMs do not co-cluster. (C) Node and edge key. Protein families are indicated by node shape and border color (left). Edges that represent interactions between proteins are colored according to interaction type (right); PTM correlations edges are at the bottom of the list. Edges that connect proteins to their PTMs in combined CFN/CCCN graphs are black. (PDF) [file pcbi.1010690.s002.pdf]

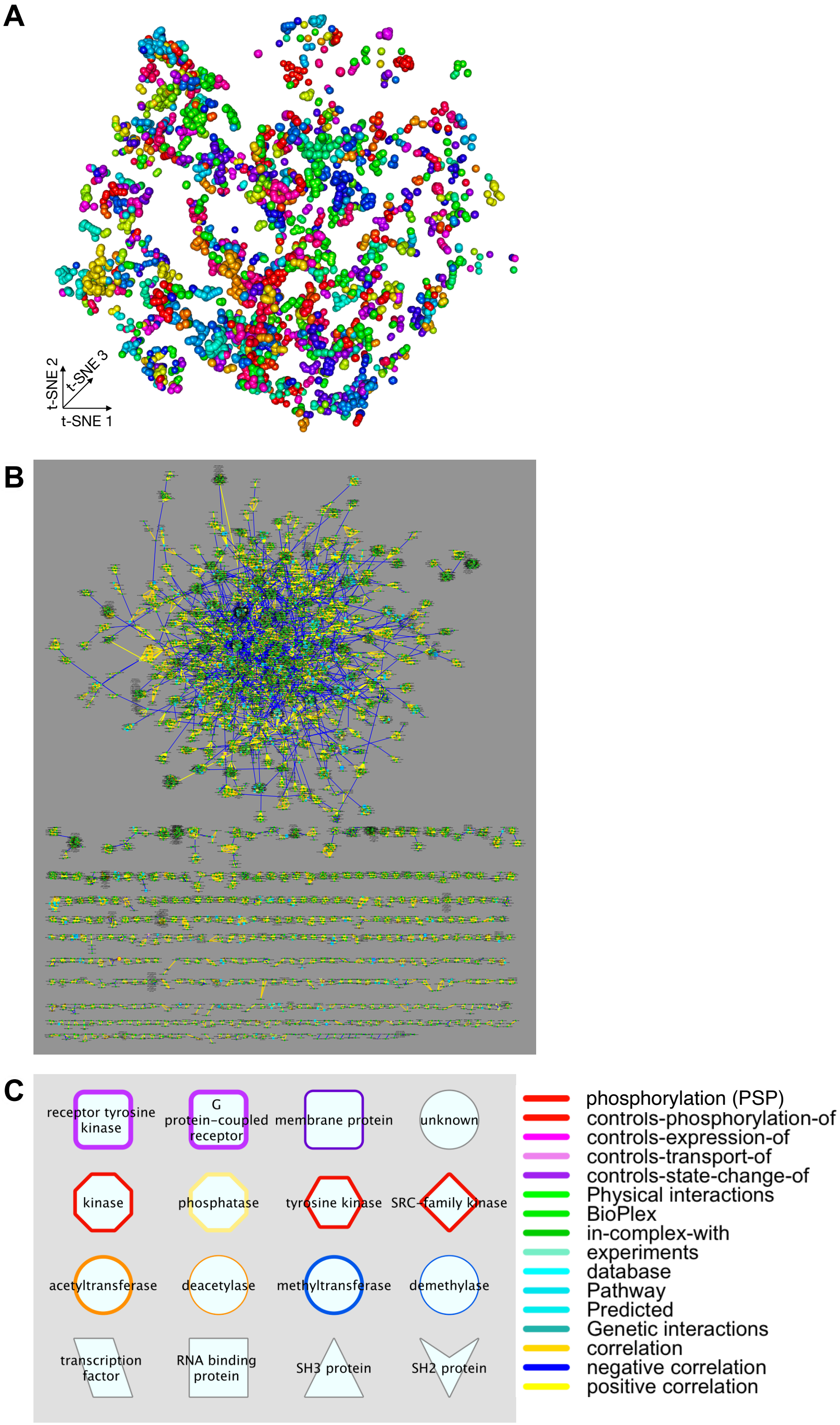

Figure S2. *Construction of the co-cluster correlation network (CCCN).* (A) Example three dimensional t-SNE embedding of PTM data using Spearman-Euclidean dissimilarity (SED) from PTM data. Co-clustered PTMs are close to one another and share the same color. (B) CCCN of all PTMs. Yellow edges are positive correlation between co-clustered PTMs; blue edges are negative correlations, negative correlations < -0.5 among different PTMs on the same protein are included even if these PTMs do not co-cluster. (C) Node and edge key. Protein families are indicated by node shape and border color (left). Edges that represent interactions between proteins are colored according to interaction type (right); PTM correlations edges are at the bottom of the list. Edges that connect proteins to their PTMs in combined CFN/CCCN graphs are black.
