# Supplementary material for: Network models of protein phosphorylation, acetylation, and ubiquitination connect metabolic and cell signaling pathways in lung cancer
Source: PLoS Comput Biol. 2023 Mar 30;19(3):e1010690. doi: 10.1371/journal.pcbi.1010690 (PMC10089347; doi:10.1371/journal.pcbi.1010690)
Supplement: S3 Fig — (A) Heatmap showing PTM log2 fold changes (key below A) on all RTK and SFK PTMs, sorted by hierarchical clustering (dendrogram at left). (B-D) CCCN interactions among RTK PTMs (left) and SFK PTMs (right). Node size and color represents log2 fold change (bar under B): blue is down-regulated; yellow up-regulated, for cells treated with erlotinib (B), crizotinib (C), and dasatinib (D). Node border and shape and edge colors are defined in S2C Fig. Edges connecting proteins to their PTMs were colored light grey for clarity. (PDF) [file pcbi.1010690.s003.pdf]

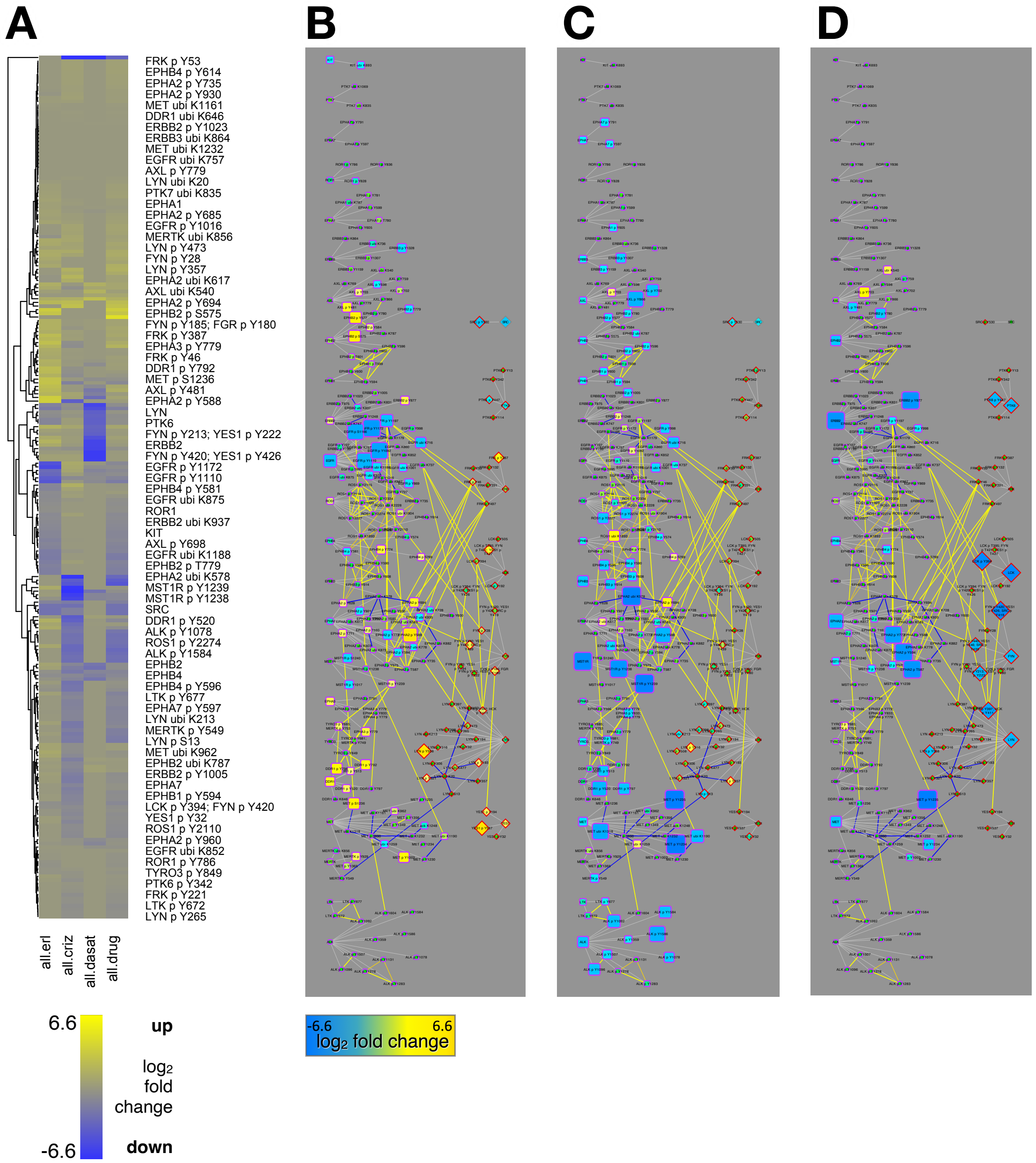

Figure S3. ***TKI effects on RTK and SFK PTMs.*** (A) Heatmap showing PTM $\log_2$ fold changes (key below A) on all RTK and SFK PTMs, sorted by hierarchical clustering (dendrogram at left). (B-D) CCCN interactions among RTK PTMs (left) and SFK PTMs (right). Node size and color represents log2 fold change (bar under B): blue is down-regulated; yellow up-regulated, for cells treated with erlotinib (B), crizotinib (C), and dasatinib (D). Node border and shape and edge colors are defined in Figure S2C. Edges connecting proteins to their PTMs were colored light grey for clarity.
