# Supplementary material for: Network models of protein phosphorylation, acetylation, and ubiquitination connect metabolic and cell signaling pathways in lung cancer
Source: PLoS Comput Biol. 2023 Mar 30;19(3):e1010690. doi: 10.1371/journal.pcbi.1010690 (PMC10089347; doi:10.1371/journal.pcbi.1010690)
Supplement: S4 Fig — (A) CFN of PPIs filtered by co-clustered PTMs. (B) Combined CFN and CCCN. Node shape and outline color, and edge colors, are defined in S2C Fig. (PDF) [file pcbi.1010690.s004.pdf]

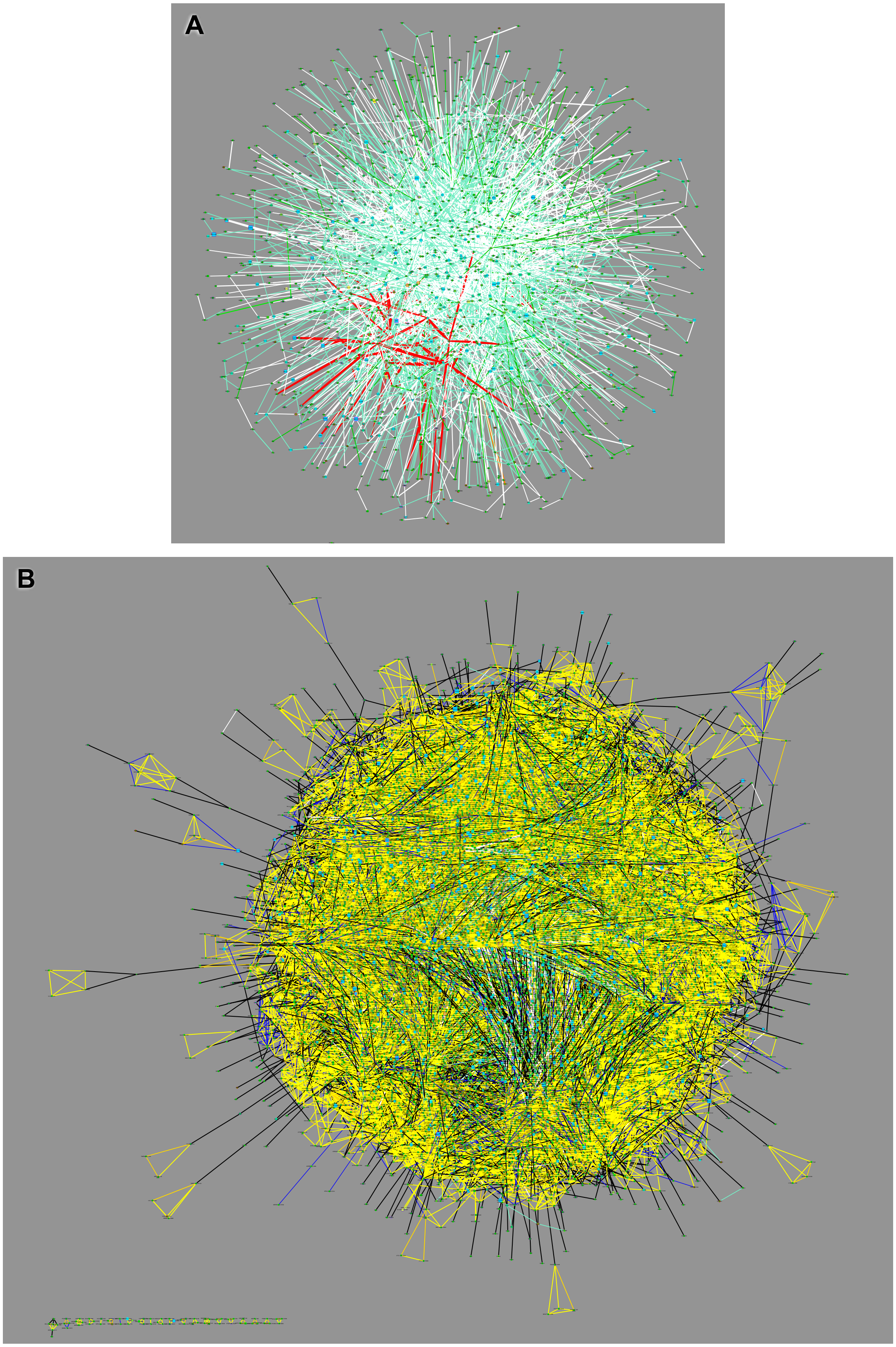

Figure S4. ***Cluster-filtered network (CFN) and combined CFN/CCCN.*** (A) CFN of PPIs filtered by co-clustered PTMs. (B) Combined CFN and CCCN. Node shape and outline color, and edge colors, are defined in Figure S2C.
