# Supplementary material for: Network models of protein phosphorylation, acetylation, and ubiquitination connect metabolic and cell signaling pathways in lung cancer
Source: PLoS Comput Biol. 2023 Mar 30;19(3):e1010690. doi: 10.1371/journal.pcbi.1010690 (PMC10089347; doi:10.1371/journal.pcbi.1010690)
Supplement: S5 Fig — Heatmap depicting, for each pair of samples, the Jaccard similarity (intersection divided by union) of the sets of genes comprising the shortest paths in the CFN connecting genes with significantly changed PTMs to the mutant driver protein/drug target. PTMs with a fold-change of at least 2.25 in drug-treated vs. control cells were defined as significantly changed. Drug/driver protein pairs were as follows: Crizotinib-ALK, Erlotinib-EGFR, Afatinib-ERBB2/HER2. (PDF) [file pcbi.1010690.s005.pdf]

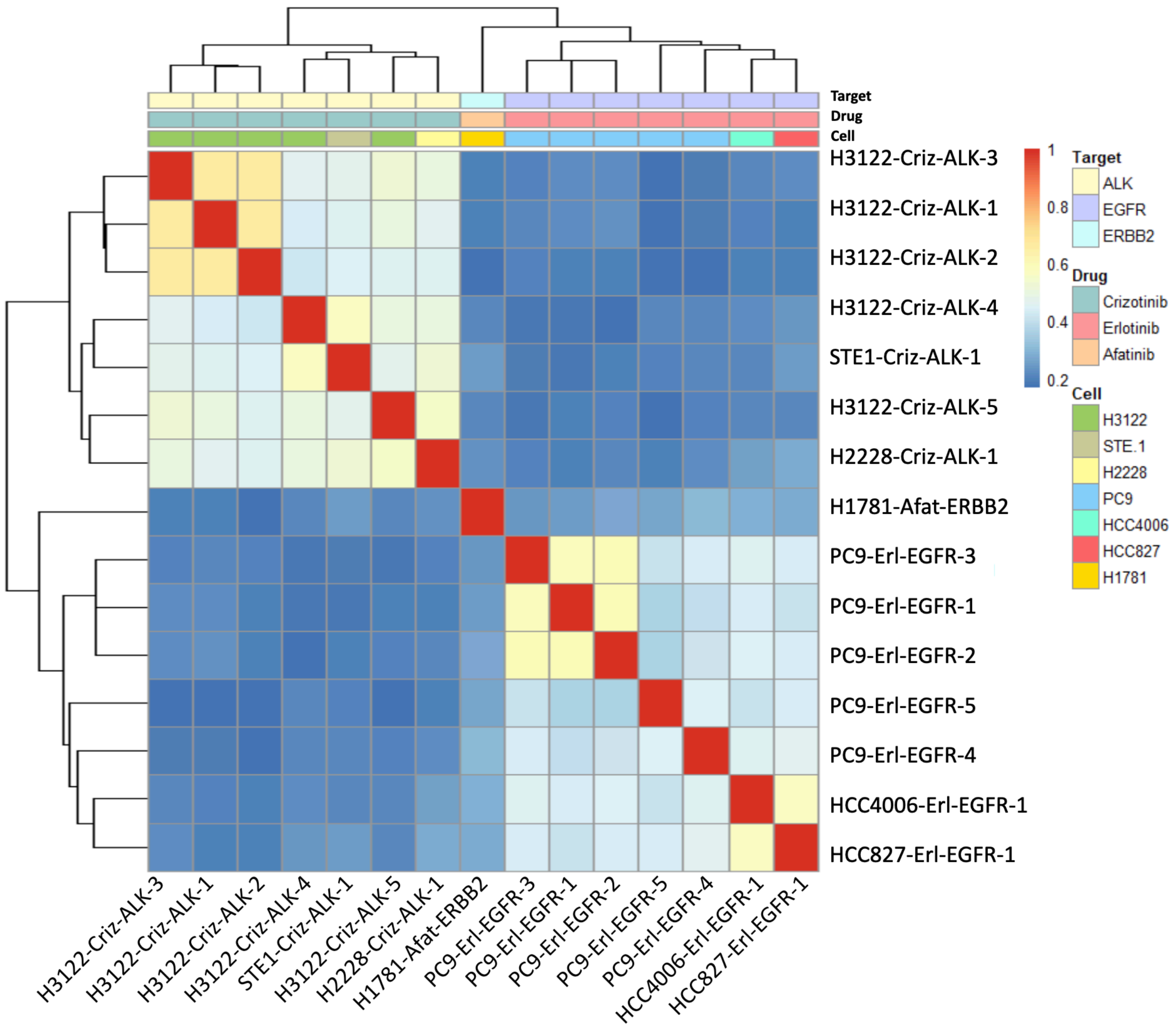

Figure S5. ***Comparison of shortest paths networks from drug targets to drug-affected sites.*** Heatmap depicting, for each pair of samples, the Jaccard similarity (intersection divided by union) of the sets of genes comprising the shortest paths in the CFN connecting genes with significantly changed PTMs to the mutant driver protein/drug target. PTMs with a fold-change of at least 2.25 in drug-treated vs. control cells were defined as significantly changed. Drug/driver protein pairs were as follows: Crizotinib-ALK, Erlotinib-EGFR, Afatinib-ERBB2/HER2.
