# Supplementary material for: Network models of protein phosphorylation, acetylation, and ubiquitination connect metabolic and cell signaling pathways in lung cancer
Source: PLoS Comput Biol. 2023 Mar 30;19(3):e1010690. doi: 10.1371/journal.pcbi.1010690 (PMC10089347; doi:10.1371/journal.pcbi.1010690)
Supplement: S6 Fig — Pathway-pathway interactions were defined based on PTM clustering, BioPlanet Jaccard similarity, or GO similarity (see Methods). (A) PTM cluster weight was poorly correlated with the BioPlanet Jaccard similarity (R2 = 0.02037). (B) GO similarity (not including genes in common between pathways) was moderately correlated with BioPlanet Jaccard similarity (R2 = 0.2208). (C) PTM cluster weight vs. GO similarity (R2 = 0.1336). Although 13% of pathway pair edges had zero GO similarity (points at 0 on the x-axis of the graph), these edges represent only a small fraction (0.34%) of the total PTM cluster weight in the network. Without these edges of zero GO similarity weight, the the R2 correlation between PTM cluster weight and GO similarity was 0.1508. (D) Same as C but excluding pathway pairs that have one or more genes in common (i.e., only interactions with Jaccard similarity = 0 were plotted; R2 = 0.06078). (PDF) [file pcbi.1010690.s006.pdf]

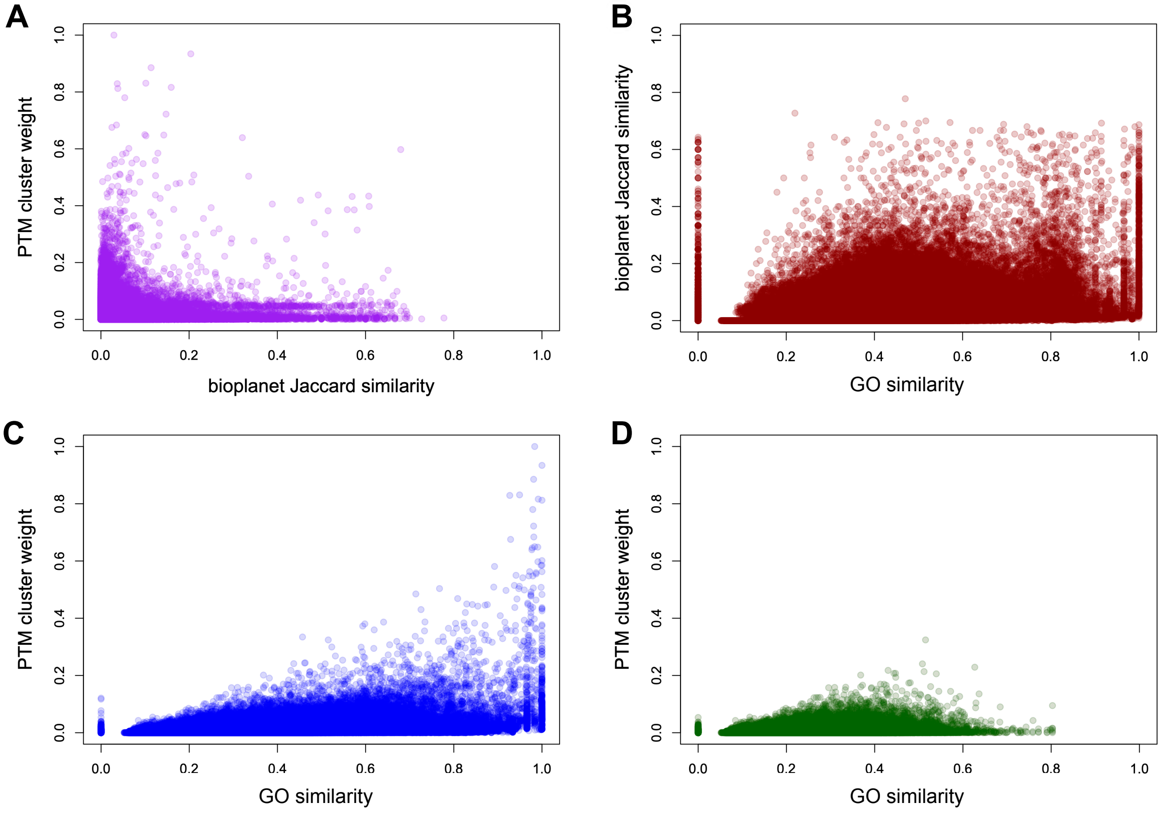


**Figure S6.** ***Comparison of PTM cluster weight with Jaccard similarity and Gene Ontology (GO) Biological Process similarity for the BioPlanet Pathway Crosstalk Network.*** Pathway-pathway interactions were defined based on PTM clustering, BioPlanet Jaccard similarity, or GO similarity (see Methods). (A) PTM cluster weight was poorly correlated with the BioPlanet Jaccard similarity ($R^2$ = 0.02037). (B) GO similarity (not including genes in common between pathways) was moderately correlated with BioPlanet Jaccard similarity ($R^2$ = 0.2208). (C) PTM cluster weight vs. GO similarity ($R^2$ = 0.1336). Although 13% of pathway pair edges had zero GO similarity (points at 0 on the x-axis of the graph), these edges represent only a small fraction (0.34%) of the total PTM cluster weight in the network. Without these edges of zero GO similarity weight, the the $R^2$ correlation between PTM cluster weight and GO similarity was 0.1508. (D) Same as C but excluding pathway pairs that have one or more genes in common (i.e., only interactions with Jaccard similarity = 0 were plotted; $R^2$ = 0.06078).
