# Supplementary material for: Network models of protein phosphorylation, acetylation, and ubiquitination connect metabolic and cell signaling pathways in lung cancer
Source: PLoS Comput Biol. 2023 Mar 30;19(3):e1010690. doi: 10.1371/journal.pcbi.1010690 (PMC10089347; doi:10.1371/journal.pcbi.1010690)
Supplement: S7 Fig — PCN filtered to show pathway pairs with strong PTM cluster weight (> 0.05, purple edges) and no genes in common (Jaccard similarity = 0). 997 out of 455988 pathway-pathway edges are shown. The pathways EGF/EGFR signaling pathway, Glycolysis and gluconeogenesis, and Transmembrane transport of small molecules are highlighted in yellow. (PDF) [file pcbi.1010690.s007.pdf]

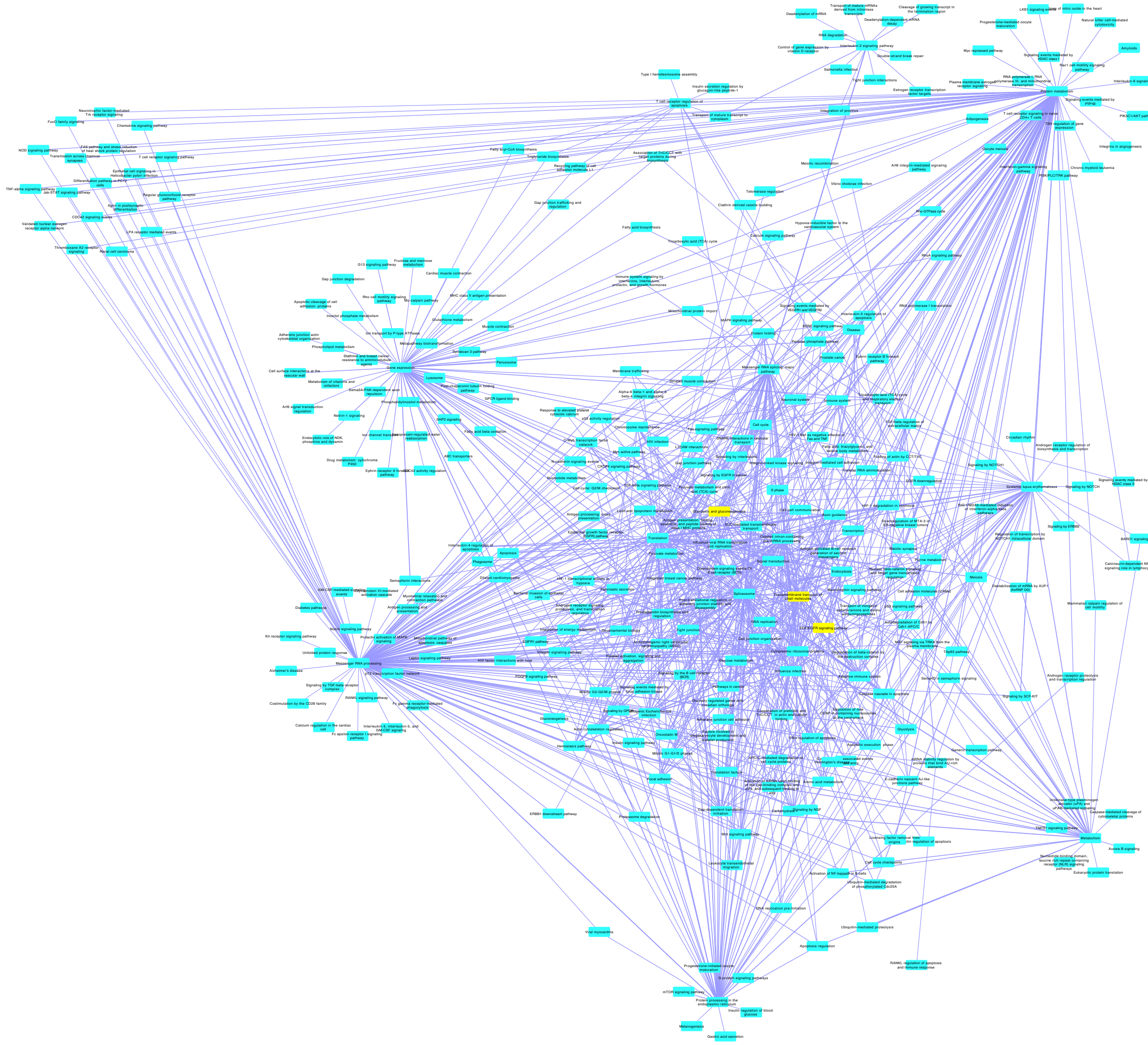

Figure S7. *Pathway Crosstalk Network (PCN) strongest cluster-based associations*. PCN filtered to show pathway pairs with strong PTM cluster weight (> 0.05, purple edges) and no genes in common (Jaccard similarity = 0). 997 out of 455988 pathway-pathway edges are shown. The pathways EGF/EGFR signaling pathway, Glycolysis and gluconeogenesis, and Transmembrane transport of small molecules are highlighted in yellow.
