# Supplementary material for: Network models of protein phosphorylation, acetylation, and ubiquitination connect metabolic and cell signaling pathways in lung cancer
Source: PLoS Comput Biol. 2023 Mar 30;19(3):e1010690. doi: 10.1371/journal.pcbi.1010690 (PMC10089347; doi:10.1371/journal.pcbi.1010690)
Supplement: S8 Fig — PTM clusters containing transporters and EGFR. (A, B, C) ABC transporter PTMs and CFN pathways connecting ABC transporters to the TKI targets EGFR, ALK, and MET. (D, E, F) PTM clusters that contain EGF/EGFR and transmembrane transporter pathway PTMs. Node size and color (scale bar in A) indicates response to erlotinib (A, D), crizotinib (B, E), and dasatinib (C, F). Node border and shape and edge colors are defined in S2C Fig. (PDF) [file pcbi.1010690.s008.pdf]

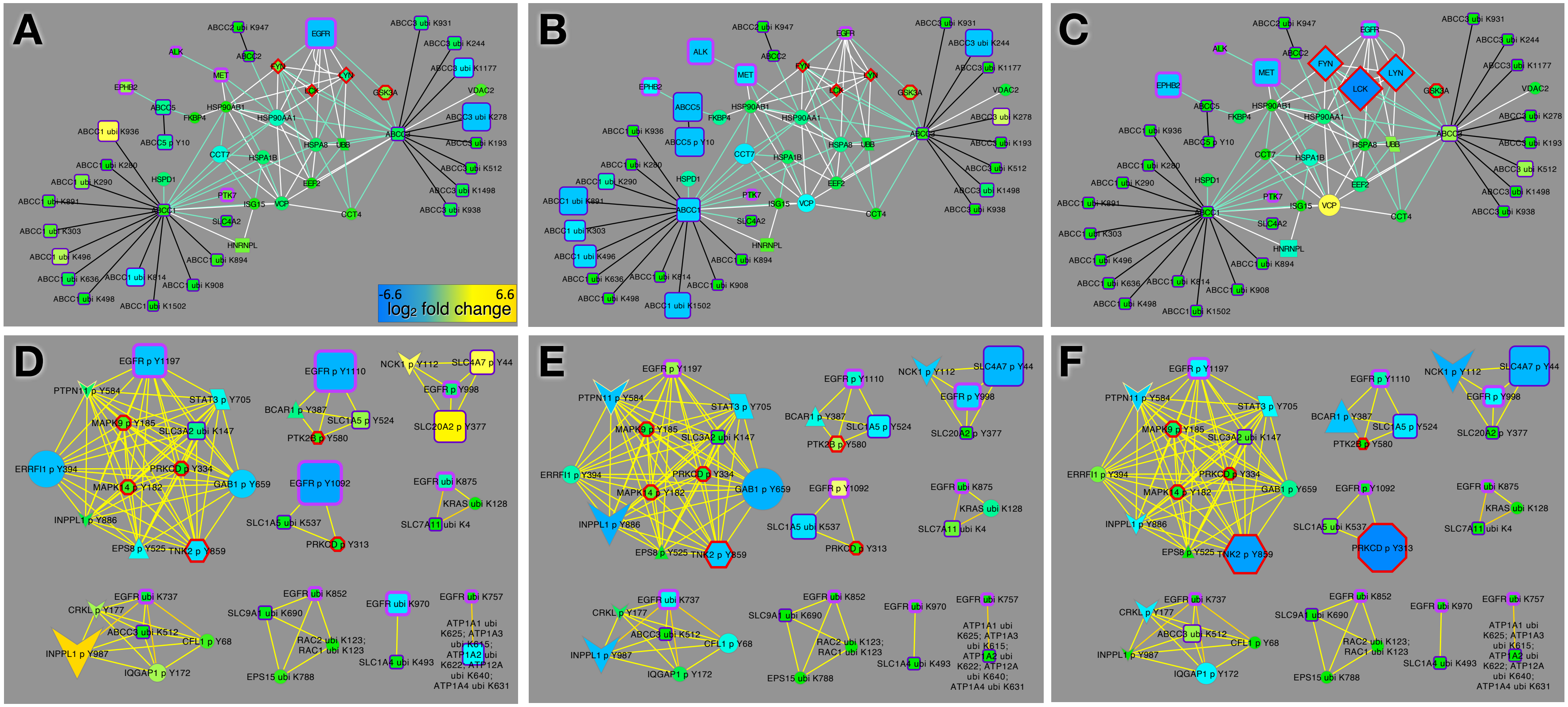

Figure S8. *Response of EGFR signaling and transporter PTMs to TKIs.* PTM clusters containing transporters and EGFR. (A, B, C) ABC transporter PTMs and CFN pathways connecting ABC transporters to the TKI targets EGFR, ALK, and MET. (D, E, F) PTM clusters that contain EGF/EGFR and transmembrane transporter pathway PTMs. Node size and color (scale bar in A) indicates response to erlotinib (A, D), crizotinib (B, E), and dasatinib (C, F). Node border and shape and edge colors are defined in Figure S2C.
