# Supplementary material for: Network models of protein phosphorylation, acetylation, and ubiquitination connect metabolic and cell signaling pathways in lung cancer
Source: PLoS Comput Biol. 2023 Mar 30;19(3):e1010690. doi: 10.1371/journal.pcbi.1010690 (PMC10089347; doi:10.1371/journal.pcbi.1010690)
Supplement: S9 Fig — Direct interactions in the CFN between members of the EGF/EGFR signaling pathway and the Transmembrane transport of small molecules pathway (A) and Glycolysis and gluconeogenesis pathway (B). PTMs that were significantly changed (>2.25-fold) in response to crizotinib in H3122 cells are shown. Node border and shape and edge colors are defined in S2C Fig. (PDF) [file pcbi.1010690.s009.pdf]

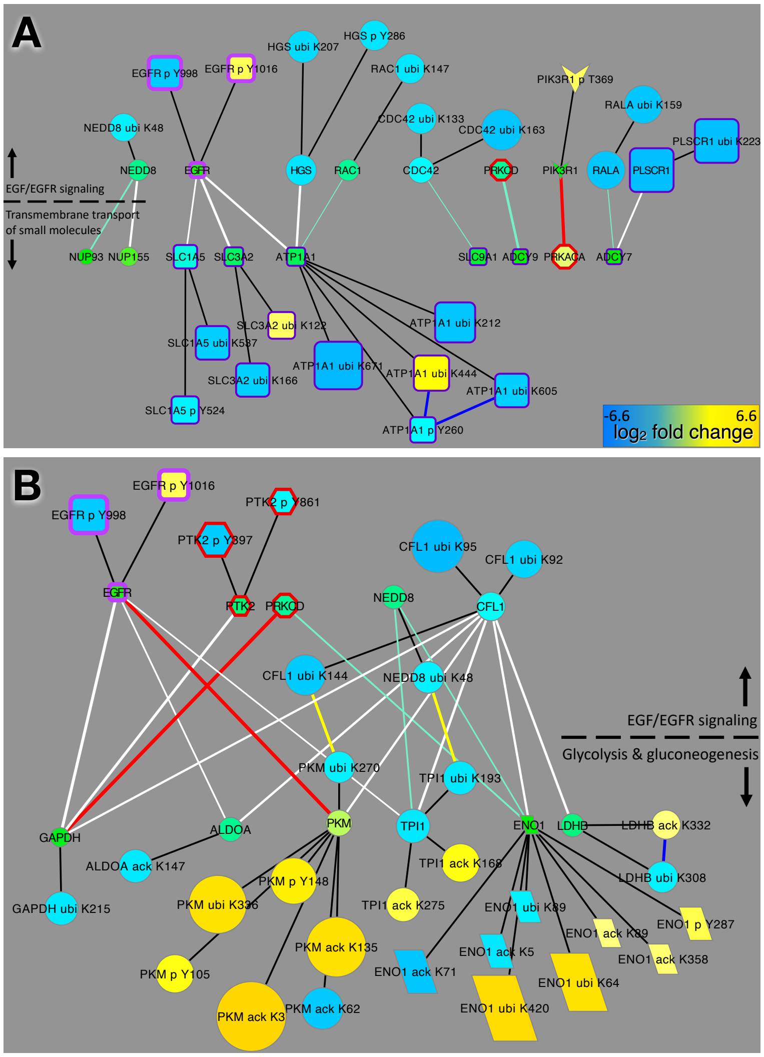

Figure S9. *Crizotinib-affected PTMs linked to EGFR signaling*. Direct interactions in the CFN between members of the EGF/EGFR signaling pathway and the Transmembrane transport of small molecules pathway (A) and Glycolysis and gluconeogenesis pathway (B). PTMs that were significantly changed (>2.25-fold) in response to crizotinib in H3122 cells are shown. Node border and shape and edge colors are defined in Figure S2C.
