# Supplementary material for: Network models of protein phosphorylation, acetylation, and ubiquitination connect metabolic and cell signaling pathways in lung cancer
Source: PLoS Comput Biol. 2023 Mar 30;19(3):e1010690. doi: 10.1371/journal.pcbi.1010690 (PMC10089347; doi:10.1371/journal.pcbi.1010690)
Supplement: S10 Fig — (A) Combined CFN/CCCN showing composite shortest paths from the BioPlanet pathways EGF/EGFR signaling pathway (top) and Glycolysis and gluconeogenesis (bottom), graphed as in Fig 3. (B) Same as A but showing CFN edges only. (C) “Mutual friends” (center row) defined as proteins that connect to at least one member of both pathways in the CFN. Node size and color represents log2 fold change (bar in B) for all TKIs (A, B) and erlotinib (C). (PDF) [file pcbi.1010690.s010.pdf]

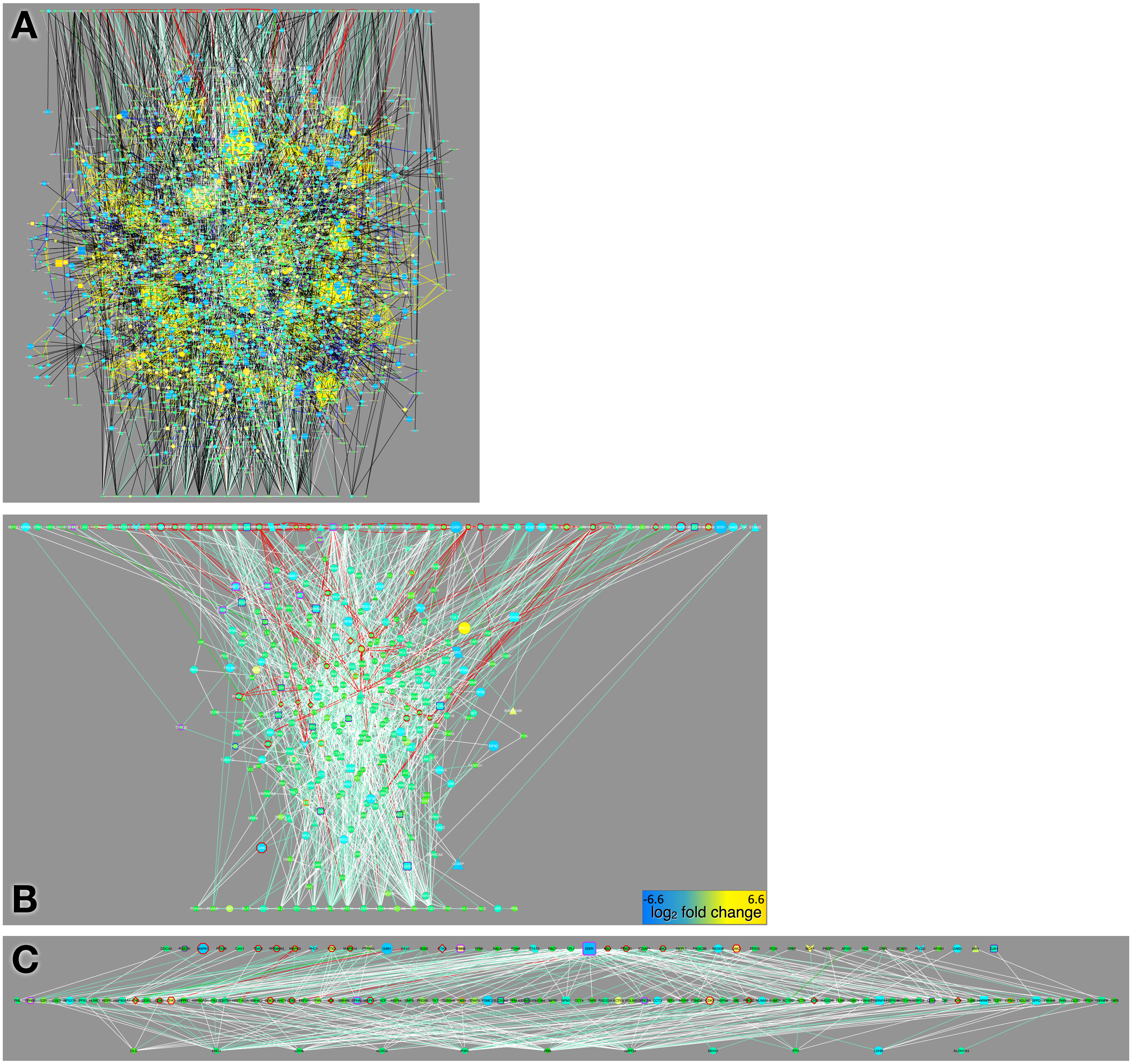

Figure S10. *EGFR glycolysis networks*. (A) Combined CFN/CCCN showing composite shortest paths from the BioPlanet pathways EGF/EGFR signaling pathway (top) and Glycolysis and gluconeogenesis (bottom), graphed as in Figure 3. (B) Same as A but showing CFN edges only. (C) “Mutual friends” (center row) defined as proteins that connect to at least one member of both pathways in the CFN. Node size and color represents $\log_2$ fold change (bar in B) for all TKIs (A, B) and erlotinib (C).
