# Supplementary material for: Network models of protein phosphorylation, acetylation, and ubiquitination connect metabolic and cell signaling pathways in lung cancer
Source: PLoS Comput Biol. 2023 Mar 30;19(3):e1010690. doi: 10.1371/journal.pcbi.1010690 (PMC10089347; doi:10.1371/journal.pcbi.1010690)
Supplement: S11 Fig — (A) CFN for proteins with PTM sites in Cluster A. Node size and color indicates the log2 ratio of PTM abundance (averaged over all PTMs for a protein) in dasatinib treated vs. untreated cells. Node border and shape and edge colors are defined in S2C Fig. (B) Heatmaps showing TKI responses of PTM sties in cluster A. Left panel: Sites in cluster A with drug-treated to control ratios that were at least 2.25-fold higher in drug-treated cells or at least 2.25-fold lower in drug-treated cells are indicated in yellow and blue, respectively. Black indicates sites that were below threshold. Right panel: the drug-treated to control ratio (median of samples in each group) for each PTM site is shown. Sample groups are as in Fig 5. (PDF) [file pcbi.1010690.s011.pdf]

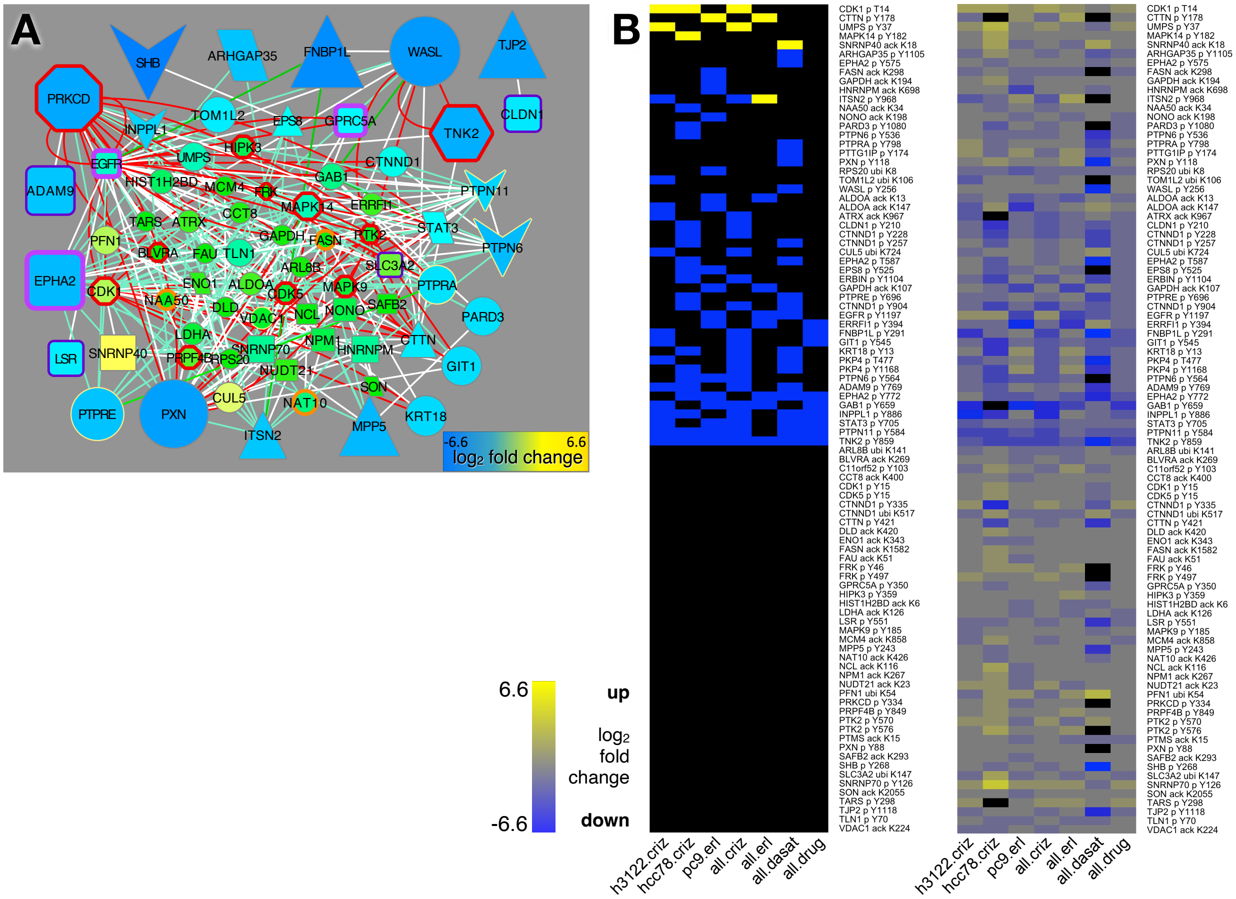

Figure S11. ***Drug-affected PTMs in cluster A.*** (A) CFN for proteins with PTM sites in Cluster A. Node size and color indicates the $\log_2$ ratio of PTM abundance (averaged over all PTMs for a protein) in dasatinib treated vs. untreated cells. Node border and shape and edge colors are defined in Figure S2C. (B) Heatmaps showing TKI responses of PTM sties in cluster A. Left panel: Sites in cluster A with drug-treated to control ratios that were at least 2.25-fold higher in drug-treated cells or at least 2.25-fold lower in drug-treated cells are indicated in yellow and blue, respectively. Black indicates sites that were below threshold. Right panel: the drug-treated to control ratio (median of samples in each group) for each PTM site is shown. Sample groups are as in Figure 5.
