# Supplementary material for: Network models of protein phosphorylation, acetylation, and ubiquitination connect metabolic and cell signaling pathways in lung cancer
Source: PLoS Comput Biol. 2023 Mar 30;19(3):e1010690. doi: 10.1371/journal.pcbi.1010690 (PMC10089347; doi:10.1371/journal.pcbi.1010690)
Supplement: S12 Fig — Heatmaps showing TKI responses of PTMs in cluster B. Left panel: Sites in cluster B with drug-treated to control ratios that were at least 2.25-fold higher in drug-treated cells or at least 2.25-fold lower in drug-treated cells are indicated in yellow and blue, respectively. Black indicates sites that were below threshold. Right panel: the drug-treated to control ratio (median of samples in each group) for each PTM site is shown (see key at bottom; black represents missing data). Sample groups are as in Fig 5. (PDF) [file pcbi.1010690.s012.pdf]

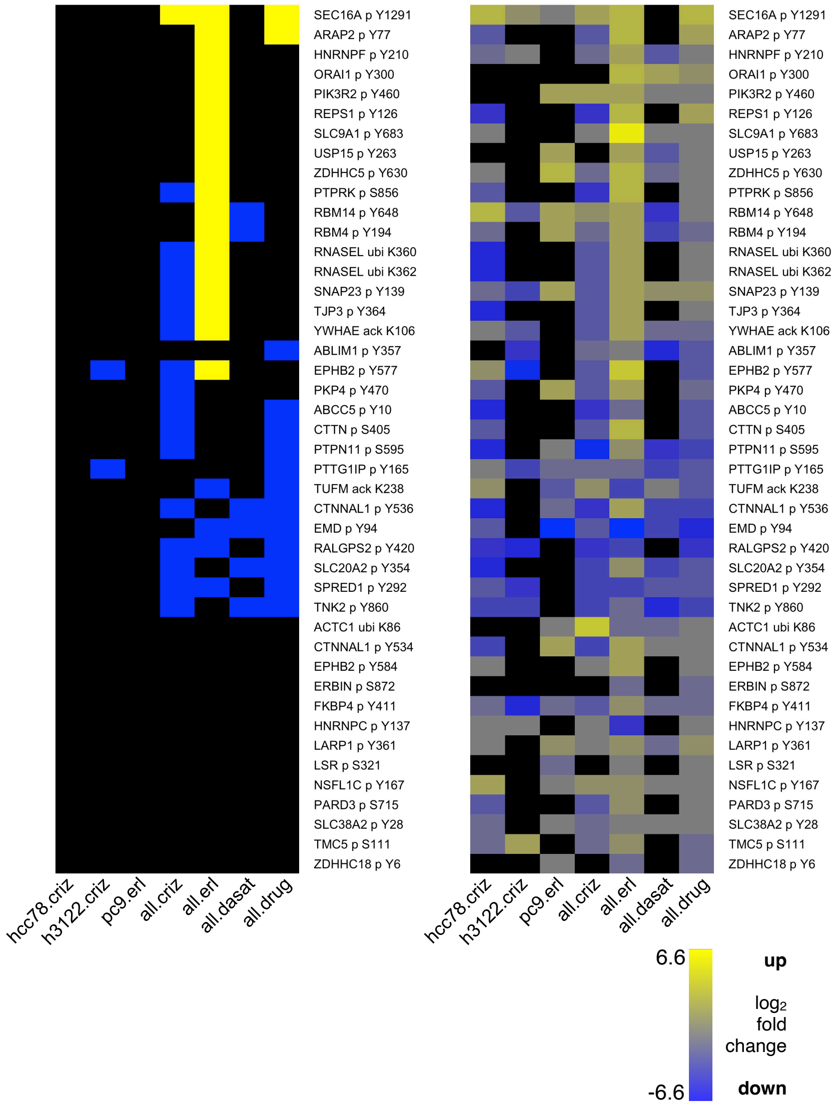

Figure S12. *Drug-affected PTMs in cluster B*. Heatmaps showing TKI responses of PTMs in cluster B. Left panel: Sites in cluster B with drug-treated to control ratios that were at least 2.25-fold higher in drug-treated cells or at least 2.25-fold lower in drug-treated cells are indicated in yellow and blue, respectively. Black indicates sites that were below threshold. Right panel: the drug-treated to control ratio (median of samples in each group) for each PTM site is shown (see key at bottom; black represents missing data). Sample groups are as in Figure 5.
