# Supplementary material for: Network models of protein phosphorylation, acetylation, and ubiquitination connect metabolic and cell signaling pathways in lung cancer
Source: PLoS Comput Biol. 2023 Mar 30;19(3):e1010690. doi: 10.1371/journal.pcbi.1010690 (PMC10089347; doi:10.1371/journal.pcbi.1010690)
Supplement: S13 Fig — CFN for proteins with PTM sites in Cluster C. Note that there are fewer nodes than there are PTM proteins in the cluster because some of the cluster proteins did not have any physical interactions with each other in the PPI databases used for CFN construction. Node size and color indicates the log2 ratio of PTM abundance (averaged over all PTMs for a protein) in PC9 erlotinib treated vs. control cells. Node border and shape and edge colors are defined in S2C Fig. (PDF) [file pcbi.1010690.s013.pdf]

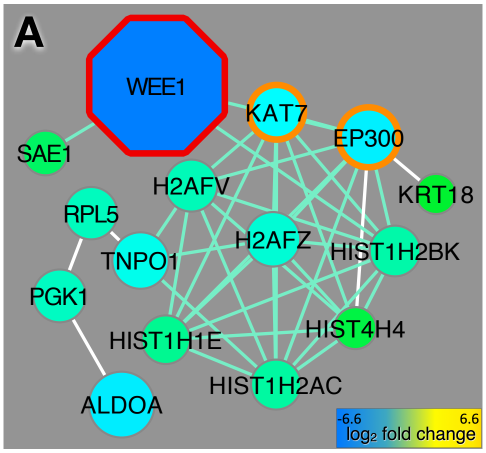

Figure S13. *Drug-affected PTMs involved in transcription regulation*. CFN for proteins with PTM sites in Cluster C. Note that there are fewer nodes than there are PTM proteins in the cluster because some of the cluster proteins did not have any physical interactions with each other in the PPI databases used for CFN construction. Node size and color indicates the log2 ratio of PTM abundance (averaged over all PTMs for a protein) in PC9 erlotinib treated vs. control cells. Node border and shape and edge colors are defined in Figure S2C.
