# Supplementary material for: Network models of protein phosphorylation, acetylation, and ubiquitination connect metabolic and cell signaling pathways in lung cancer
Source: PLoS Comput Biol. 2023 Mar 30;19(3):e1010690. doi: 10.1371/journal.pcbi.1010690 (PMC10089347; doi:10.1371/journal.pcbi.1010690)
Supplement: S14 Fig — (A) Shared core matrix. The CST-CFN from a previous study [15] is plotted on the x-axis; the CFN from this study on the y-axis. The numbers indicate the number of proteins in common in each pair of cores. Cells are colored yellow, orange and red by the number of common proteins. Note that the CST-CFN lacks an 8th core, and the 9th and 10th cores represent a clique of interconnected ribosomal proteins. (B) Number of proteins in each core found in the CFN from this study (left) and the CST-CFN (right). (C) PTMs detected in this study from 43 proteins that appear in the high cores of both CFNs (Fig 7) graphed as a CCCN using data from this study. Node size and color reflects PTM changes in cells treated with all TKIs. (PDF) [file pcbi.1010690.s014.pdf]

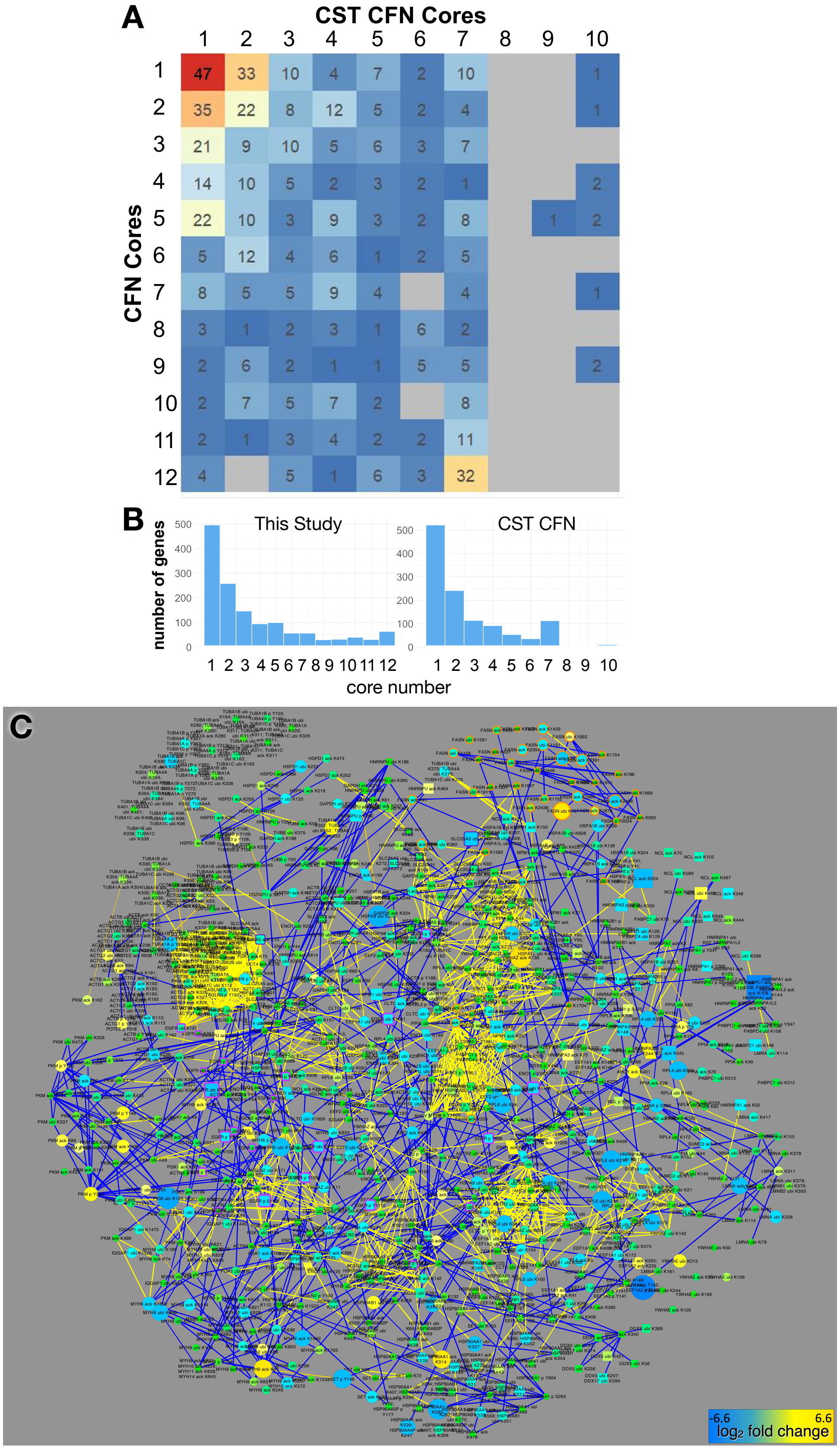

Figure S14. *Comparison of networks (CFNs) from different lung cancer PTM data sets.*(A) Shared core matrix. The CST-CFN from a previous study [15] is plotted on the x-axis; the CFN from this study on the y-axis. The numbers indicate the number of proteins in common in each pair of cores. Cells are colored yellow, orange and red by the number of common proteins. Note that the CST-CFN lacks an 8th core, and the 9th and 10th cores represent a clique of interconnected ribosomal proteins. (B) Number of proteins in each core found in the CFN from this study (left) and the CST-CFN (right). (C) PTMs detected in this study from 43 proteins that appear in the high cores of both CFNs (Figure 7) graphed as a CCCN using data from this study. Node size and color reflects PTM changes in cells treated with all TKIs.
