# Supplementary material for: Network models of protein phosphorylation, acetylation, and ubiquitination connect metabolic and cell signaling pathways in lung cancer
Source: PLoS Comput Biol. 2023 Mar 30;19(3):e1010690. doi: 10.1371/journal.pcbi.1010690 (PMC10089347; doi:10.1371/journal.pcbi.1010690)
Supplement: S1 Text — (DOCX) [file pcbi.1010690.s015.docx]

**Supplementary Text S1**

**Comparison of Shortest Paths Subnetworks**

We compared the shortest paths subnetworks connecting the drug target (i.e., the mutated cancer-driver protein) in each sample to the proteins whose PTMs changed in response to drug treatment. For H3122, H2228, and STE1 samples, the driver kinase/drug target was ALK; for PC9, HCC4006, and HCC827 samples the driver kinase/drug target was EGFR; and for the H1781 sample the driver kinase/drug target was ERBB2/HER2. H2286 and H366 samples were not included because the driver kinase in these cells, DDR2, was not included in the CFN. Similarly, HCC78 cells were not included because the driver kinase, ROS1, was not included in the CFN. For each drug/cell-line combination, we selected the proteins that had at least one PTM site that was at least 2.25-fold increased or decreased in drug-treated cells relative to control cells. Next, we identified all shortest paths connecting those proteins to the drug target (i.e., the mutated cancer-driver protein). We calculated the Jaccard similarity (intersection divided by union) of the proteins in the subnetworks for each pair of samples and plotted the results in a heatmap using heatmap.2 from the [gplots package (version 3.1.3)](https://www.rdocumentation.org/packages/gplots/versions/3.1.3) in R. As expected, replicates of the same drug/cell-line combination have the highest similarity, followed by different cell lines treated with same drug. Samples treated with different drugs had the lowest similarity (Figure S5). These results suggest that despite the inherent noisiness of the underlying PTM proteomic data, the CFN can reproducibly capture signaling responses to TKIs.

**Comparison of PTM Cluster Weight to Other Measures of Pathway Similarity**

We compared the PTM cluster weights to two other measures of pathway-pathway similarity: i) Jaccard similarity (intersection/union of pathway genes), which captures the extent to which a pair of pathways has genes in common, and ii) Gene Ontology (GO) similarity, which measures the extent to which genes in the two pathways are annotated with common GO Biological Process terms (see Methods) [1]. The PTM cluster weight was poorly correlated with the Jaccard similarity (R^2^ = 0.02, Figure S6A), which indicates this approach is not simply recapitulating pathway relationships based on genes in common. GO similarity had a moderate correlation with Jaccard similarity (R^2^ = 0.22; Figure S6B). Overall, the correlation between PTM cluster weight and GO similarity was low (R^2^ = 0.13), though pathway pairs that had high PTM cluster weight did tend to have higher GO similarity (Figure S6C). The converse is not true, however; there were pathways with high GO similarity that had low PTM cluster weight, which probably reflects that our data do not represent complete coverage of all PTMs in all cells due to limitations in the enrichment of these selected PTMs, the digestion enzyme, and mass spectrometry detection. Comparing the plot of PTM cluster weight vs. GO similarity for all pathway pairs (Figure S6C) to the plot that includes only those pathway pairs that had zero Jaccard similarity (no genes in common; Figure S6D) indicates that most of the pathway pairs with very high GO similarity had genes in common, as did many of the pathway pairs with the highest PTM cluster weight. Pathway pairs with high PTM cluster weights that had no genes in common tended to have intermediate GO similarity (Figure S6D). These comparisons support the hypothesis that the PTM clusters return biologically relevant information that is complementary to, and independent of, common genes or common GO annotation, and that the PTM cluster weight will be useful for filtering relationships between pathways.

**CCCN Clusters Enriched for Drug-Affected PTMs**

We defined drug-affected sites to be sites whose median abundance ratio in drug-treated vs. control cells was at least 2.25-fold (up or down) in samples grouped by cell type and drug treatment (called sample groups, defined in Methods). The complete list of drug-affected sites in each sample group can be found in Table S4. Out of 818 clusters, there were 137 that were enriched for drug-affected sites in at least one cell-line/drug combination, (corrected p-value < 0.05, Fisher’s Exact Test), including 91 clusters enriched in more than one sample group (Table S5). There were multiple enriched clusters for each of the sample groups (range: 11-64 clusters, mean: 40 clusters). The non-random distribution of drug-affected sites among clusters in a variety of sample groups confirms that our clustering method produced clusters that respond to perturbations in a coordinated manner and may represent biological modules.

**Network Comparison Using k-core Analysis**

The CST-CFN and our CFN networks shared 583 common nodes (proteins) and 239 edges (out of 3504 edges in the union of the two networks). Edge density values were 0.04 in both networks, which indicate high sparsity. The highest degree was 124 in our CFN (HSP90AA1) and 54 in CST CFN (EEF2). The distance between PTM pairs showed similar distribution on both networks; most node pairs were 4-edges distant from each other. Our CFN had more nodes than the CST-CFN, but the network diameter of our CFN was smaller than the CST-CFN. This indicates that the network had shortened its diameter with increasing node count (similar to real-life social network dynamics). We performed a hub analysis [2] and found that the CST-CFN contained more hub nodes than our CFN. That is, the CST-CFN had more nodes that connect distant parts of the network. This is surprising because our CFN had ten times more nodes in its higher cores; thus, their high-coreness must be due to connections to each other, which further indicates dense subgraphs.

Our CFN had 12 cores; we considered cores 11-12 to be high cores (Figure S14A, B). The CST-CFN had 10 cores; we considered cores 7 and above to be high cores because the network had no nodes in its 8th core and the 9th and 10th cores were a clique of 12 interacting acetylated ribosomal protein genes (NOP58, RPL18A, RPL23A, RPL26, RPL3, RPL35, RPL5, RPS25, RPS28, RPS6, RPS9, SSR3). These vertices were not connected to the nodes in lower cores, which indicates a specialized function, namely control of translation through ribosomal protein acetylation. In our network, the high cores (≥11) contained 95 nodes (Figure S14A, B); in the CST-CFN network, the high cores (≥7) contained 125 nodes. Of the 95 nodes in the high cores (≥11) of our CFN, 76 (80%) were found in in the CST-CFN; of the 125 nodes in the high cores (≥7) of the CST-CFN, 107 (86%) were found in our CFN. There were 43 nodes in common in the high cores of both networks (cores ≥11 for our CFN and cores ≥7 for the CST-CFN); these common nodes were connected by 289 edges in our CFN and and 137 edges in the CST-CFN.

**Determination of Optimal Number of Gene Pairs (N) to Include in GOSim Score**

A crucial step in developing GOSim was to specify the number of gene-pair similarities used in the calculation of pathway-pathway similarity (N). A large *N* value penalizes large pathways, which will have many gene pairs, most of which are likely to have small similarity values, whereas a small *N* value ignores useful gene similarity information beyond the *Nth* pair. We would like to choose a value of N, such that the GOSim score reflects the “true” level of similarity between the pathways. Although we do not know “true” pathway similarity, we reasoned that pathway-pathway Jaccard similarity, which is based on the number of genes in common between two pathways might be an adequate approximation. Our GOSim calculation does not include genes in common between two pathways; however, when two pathways contain many genes in common, we might expect their remaining genes to have similar GO Biological Process (GOBP) annotations and vice versa. Therefore, we determined the goodness-of-fit (R^2^) for a linear model of GOSim vs. Jaccard Similarity for all Bioplanet pathway pairs with a non-zero cluster weight (645,709 pairs) for GOSim calculated using a range of values of N (*N* ∈ *(10, 30, 50, 100, 200))*. The results are shown in Table 1.

| N | R^2^ |
| --- | --- |
| 10 | 0.2278 |
| 30 | 0.2208 |
| 50 | 0.2095 |
| 100 | 0.1875 |
| 200 | 0.1628 |

Table 1: GOSim vs. Jaccard similarity goodness-of-fit (R^2^) for a range of numbers of gene-pairs (N) used in the GOSim calculation.

The R^2^ values decreased as N increased (more lower similarity gene-pairs used in the score). Because the R^2^ values for N=10 and N=30 were very similar, we chose N=30 because it allowed us to include more information in the GOSim score, without significantly degrading the score with many low similarity gene pairs.

# References

1. Gan M. Correlating information contents of gene ontology terms to infer semantic similarity of gene products. Comput Math Methods Med. 2014;2014:891842. Epub 20140522. doi: 10.1155/2014/891842. PubMed PMID: 24963342; PubMed Central PMCID: PMCPMC4054916.

2. Kleinberg JM. Hubs, authorities, and communities. ACM Comput Surv. 1999;31:5.
