# Supplementary material for: Network models of protein phosphorylation, acetylation, and ubiquitination connect metabolic and cell signaling pathways in lung cancer
Source: PLoS Comput Biol. 2023 Mar 30;19(3):e1010690. doi: 10.1371/journal.pcbi.1010690 (PMC10089347; doi:10.1371/journal.pcbi.1010690)
Supplement: Attachment — Submitted filename: PlosCompBiolRevisedCover.pdf [file pcbi.1010690.s024.pdf]

UNIVERSITY OF MONTANA

Division of Biological Sciences
Cellular, Molecular & Microbial Biology
University of Montana
Missoula, Montana 59812

February 9, 2023

Feixiong Cheng, Ph.D.
Guest Editor
Jian Ma
Section Editor
PLOS Computational Biology

Dear Dr. Cheng and Dr. Ma

We thank the reviewers for their thoughtful comments and for helping us to improve the manuscript. Responses to each point raised by the reviewers are provided below.

Reviewer #1

1. *The main text is very long in this manuscript, which makes the readers hard to follow. I suggest some of the main text should be removed as supplementary method;*

   We have shortened the main text by ~20%. Some of the details have been moved to a new supplementary document called Supplementary text S1.

2. *The resolution of some figures is low. They should be replaced with high resolution ones;*

   We have confirmed that all of our figures were high resolution pdf files at the time of submission. It is possible that some resolution was lost in the copies of the figures that were sent to the reviewers.

3. *The authors compared networks (CFNs) from different lung cancer PTM data sets, and reveals a common core of PPIs. The more potential application of this finding in future should be discussed.*

   We have added text to the Discussion about potential application of our common core analysis. Specifically, we note that our analysis revealed many examples of competing ubiquitination and acetylation on proteins in the common core. Thus, therapies that target enzymes involved in acetylation

and ubiquitination, and protein interactions involving recognition of these modifications, could be a valuable additional means to modulate cell signaling pathways to mitigate chemotherapy resistance.

Reviewer #2

1. *In Introduction, the author introduced the concepts and importance of Protein post-translational modifications, lung cancer and their hypothesis, making it easier to understand the study's background and targets. However, the motivation and innovation of this project is not very clearly in this part. I suggest that the motivation, innovation as well as the main contribution of this project should be summarized in the Introduction section.*

   We have revised the Introduction to make our motivation and contributions clearer. Specifically, we added the following text: "To work towards the goal of development of new therapies to circumvent or overcome drug resistance, the motivation for this study is to acquire a better understanding of how cell signaling networks are modulated by mutated kinases and TKIs in lung cancer cell lines. Our approach to examining cell signaling pathways with high resolution is to integrate PTM proteomic data, monitoring changes in multiple PTMs in response to TKIs, with protein interaction networks and biological pathways." And: "We hypothesized that different TKIs would perturb cell signaling pathways, which would be reflected by PTM changes, to reveal mechanisms unique to the drug target and common core pathways that respond to all TKIs."

2. *The authors try to use Figure 1 to illustrate the main strategy of this project. However, I don't think this figure is suitable to show the pipeline of the methods (at least it not very obviously). I suggest the authors to reorganize the Figure 1, which would make the work more understandable.*

   We have revised Figure 1 to make the logical flow clearer how our proteomic data was used in the informatics analysis and to explain the relationship among the different networks (CCCN, CFN, and PCN) that we used in the analysis.

3. *After the introduction, the network model, the results focus on the analysis of EGFR. And it seems that the network models just verify the importance of the EGFR. How we can learn from the network model is not very clear. And more statistic analysis of the network models should be provided.*

In order to broaden the analysis beyond EGFR, we have added a new figure (Figure S9) that shows the response to crizotinib in ALK-driven lung cancer cells of PTMs in the EGFR signaling, small molecular transport, and glycolysis pathways. We compare the responses to those of erlotinib-treated EGFR-driven cells. We have also added text to the Discussion to point out potential therapeutic applications of our findings (see response to Reviewer #1, point 3 above). Finally, we provide a comparison of basic network statistics for the CFN discussed in this paper and the CFN from our previous work (CST-CFN) in the new supplementary text document, Supplementary text S1 (section "Network Comparison Using k-core Analysis")

4. *There are many methods to identify the core of network, such as the mining of the dense subgraph. The authors used the k-core decomposition. And what about the result of the other methods?*

   The k-core decomposition is a method for identifying the core of a network, but it is not the only method that can be used. Other methods for identifying the core of a network include:
   1- Identifying densely connected subgraphs: A densely connected subgraph is a subgraph in which the majority of nodes are connected to a large number of other nodes. These subgraphs can be considered the core of the network because they are the most interconnected and central parts of the network.
   2- Identifying central nodes: Central nodes are nodes that have a high degree or centrality within the network. These nodes can be considered the core of the network because they play a central role in connecting other nodes.
   3-Identifying communities: Communities within a network are groups of nodes that are more highly connected to each other than they are to the rest of the network. The nodes within a community can be considered the core of that community, as they are the most closely connected and central to that group.

   It is important to note that the choice of method for identifying the core of a network will depend on the specific characteristics of the network and the research goals of the study. In this work, core decomposition provides an intuitive approach to order nodes in graphs based on hierarchical cores. This allows us to compare networks hierarchically by considering the number of neighbors only in cores. Densest subgraphs do not provide an intuitive hierarchy. Furthermore, densest subgraph methods require parameter tuning and selection that are not necessary in core decomposition. A further advantage is that decomposition is computationally cheap compared to other

clustering, centrality and subgraph discovery methods.

5. *In page 8, the authors stated that "chose one target for each drug …". Why only one target was selected? How to choose the target for more than one targets of the drug?*

   We have clarified the text to indicate that the target selected in each case was the mutant cancer-driver gene in that cell line. For example, for H3122 cells that are driven by an ELM4-ALK rearrangement, we used ALK as the crizotinib target; for H1781 cells that are driven by an ERBB2/HER2 mutation, we used ERBB2/HER2 as the afatinib target.

6. *The statement "Cluster Pathway Evidence (i,j) =…" in page 32. I suggest the authors can use the mathematical formula to describe the calculation. As well as the GeneSim, Jaccard Similarity.*

   We have revised the text to show the mathematical formulae for Cluster Pathway Evidence, GeneSim, and Jaccard Similarity, and numbered the equations.

7. *In the definition of the GoSim, the authors state that they achieved the best results with N=30. What's the meaning of the best results? Are there any other pa rameters used in the network models? And what's the influence?*

We have added the following text to the Supplementary Text section to describe the influence of the choice of N on the similarity score and to describe how we chose N=30 as the optimal value.

"A crucial step in developing GOSim was to specify the number of gene-pair similarities used in the calculation of pathway-pathway similarity (N). A large N value penalizes large pathways, which will have many gene pairs, most of which are likely to have small similarity values, whereas a small N value ignores useful gene similarity information beyond the Nth pair. We would like to choose a value of N, such that the GOSim score reflects the "true" level of similarity between the pathways. Although we do not know "true" pathway similarity, we reasoned that pathway-pathway Jaccard similarity, which is based on the number of genes in common between two pathways might be an adequate approximation. Our GOSim calculation does not include genes in common between two pathways; however, when two pathways contain many genes in common, we might expect their remaining genes to have similar GO Biological Process (GOBP) annotations

and vice versa. Therefore, we determined the goodness-of-fit (R^2) for a linear model of GOSim vs. Jaccard Similarity for all Bioplanet pathway pairs with a non-zero cluster weight (645,709 pairs) for GOSim calculated using a range of values of N (N ∈ (10, 30, 50, 100, 200)). The results are shown in Table 1.

N	R^2
10	0.2278
30	0.2208
50	0.2095
100	0.1875
200	0.1628

Table 1: GOSim vs. Jaccard similarity goodness-of-fit for a range of numbers of gene-pairs (N) used in the GOSim calculation.

The R^2 values decreased as N increased (more lower similarity gene-pairs used in the score). Because the R^2 values for N=10 and N=30 were very similar, we chose N=30 because it allowed us to include more information in the GOSim score, without significantly degrading the score with many low similarity gene pairs."

Reviewer #3

1. *On page 33-35, authors define the similarity of a pathway. This looks similar to "GO semantic similarity measurement" (https://yulab-smu.top/biomedical-knowledge-mining-book/GOSemSim.html), while I am not sure if they are the same. If so, I think that authors do not have to explain the details again but just cite it. If not – it means that authors developed this measurement from scratch relatively – then I suggested that the authors could provide more details about how they calculate the cosine similarity between two vectors with different dimensions. I just tried the "distance.cosine" from Python and "cosine" from lsa R package, and none of them can handle different lengths of vectors*.

   Our method has some similarities to GoSemSim, although ours is a more simplistic measure because it determines the similarity of two gene products based on the extent to which they have overlapping identical GO terms—it does not take advantage of the GO hierarchy. We apologize for the confusion about our calculation of cosine similarity. We intended to say that we used

vectors of length equal to the total number of GOBP terms. Each gene product was then represented by a vector of 0's and 1's, where 1 indicates the gene product is annotated with the GOBP term represented by that vector element, and 0 indicates it not. However, this approach is computationally inefficient due to the large vector sizes, which are a result of the large number of GOBP terms. Therefore, we ultimately calculated the similarity using the Otsuka–Ochiai coefficient, which is equivalent to the cosine similarity:

$$GeneSim(gene_A, gene_B) = \frac{|GOBP_A \cap GOBP_B|}{\sqrt{|GOBP_A| \times |GOBP_B|}}$$

where,
$GOBP_A$ = set of GOBP annotations for gene A
$GOBP_B$ = set of GOBP annotations for gene B

We have revised the text to correct this explanation.

2. *Some suggestions about the formatting of the equations but it is very minor*
    a. *Using either LaTeX way to format equations or plain text to format equations, but it would be better to keep it consistent across all equations.*
    b. *Add equation number so that the reader could refer to any equation in the paper easily.*

    We have converted all of our equations to LaTeX format. We have numbered the equations.

3. I suggest to add references to the R packages and other softwares mentioned in the paper, e.g., igraph (Csardi G, Nepusz T (2006). "The igraph software package for complex network research." InterJournal, Complex Systems, 1695. https://igraph.org.); MaxQuant PMID:19029910, PMID:25059623 (https://scicrunch.org/resolver/SCR_014485).

    References to software and packages such as MaxQuant, Cytoscape, RCy3, igraph, and Bioconductor have been added. (Where no references could be found, links to the online descriptions are provided.)

4. *On page 34, it is very good that the authors experimented with different N values, but it would be better for the author to release the results of this experiment in the supplementary either in table or figure as a guidance for*

reproduction or applying the method on new datasets.

See response to Reviewer #2, point #7.

Sincerely,

Mark Grimes
