# Supplementary material for: Network models of protein phosphorylation, acetylation, and ubiquitination connect metabolic and cell signaling pathways in lung cancer
Source: PLoS Comput Biol. 2023 Mar 30;19(3):e1010690. doi: 10.1371/journal.pcbi.1010690 (PMC10089347; doi:10.1371/journal.pcbi.1010690)
Supplement: Attachment — Submitted filename: PCOMPBIOL-D-22-01566_MJ_reivew.docx [file pcbi.1010690.s023.docx]

In this Ross et al. manuscript, the authors analyzed large-scale PTM data to identify cell signaling pathways affected by TKIs in ten lung cancer cell lines. They applied/developed machine learning methods to identify PTM clusters that represent functional modules in cell signaling pathways. The PTM clusters could be further used to identify protein-protein interaction (PPI) while these interactions between cell signaling pathways are active in lung cancer cells treated with anti-cancer drugs. By modeling these interactions in different levels of pathways, PPI and PTM, the authors identified previously unappreciated mechanisms of crosstalk among signaling pathways between oncogenic tyrosine kinase signaling and proteins that regulate metabolic reprogramming in lung cancer. And more importantly, their results revealed potential drug targets for combination therapy.

I also have a few minor comments regarding technical details (in no particular order).

- On page 33-35, authors define the similarity of a pathway. This looks similar to “GO semantic similarity measurement” (<https://yulab-smu.top/biomedical-knowledge-mining-book/GOSemSim.html>), while I am not sure if they are the same. If so, I think that authors do not have to explain the details again but just cite it. If not – it means that authors developed this measurement from scratch relatively – then I suggested that the authors could provide more details about how they calculate the cosine similarity between two vectors with different dimensions. I just tried the “distance.cosine” from Python and “cosine” from lsa R package, and none of them can handle different lengths of vectors.
- Some suggestions about the formatting of the equations but it is very minor:
  - Using either LaTeX way to format equations or plain text to format equations, but it would be better to keep it consistent across all equations.
  - Add equation number so that the reader could refer to any equation in the paper easily.
- I suggest to add references to the R packages and other softwares mentioned in the paper, e.g., igraph (Csardi G, Nepusz T (2006). “The igraph software package for complex network research.” InterJournal, Complex Systems, 1695. <https://igraph.org>.); MaxQuant PMID:19029910, PMID:25059623 (<https://scicrunch.org/resolver/SCR_014485>).
- On page 34, it is very good that the authors experimented with different N values, but it would be better for the author to release the results of this experiment in the supplementary either in table or figure as a guidance for reproduction or applying the method on new datasets.
